# Metabolic Syndrome and Fatal Outcomes in the Post-Stroke Event: A 5-Year Cohort Study in Cameroon

**DOI:** 10.1371/journal.pone.0060117

**Published:** 2013-04-02

**Authors:** Eric Vounsia Balti, André Pascal Kengne, Jean Valentin Fogha Fokouo, Brice Enid Nouthé, Eugene Sobngwi

**Affiliations:** 1 Diabetes Research Center, Faculty of Medicine and Pharmacy, Brussels Free University, Brussels, Belgium; 2 National Obesity Center, Yaoundé Central Hospital and Faculty of Medicine and Biomedical Sciences, University of Yaoundé 1, Yaoundé, Cameroon; 3 NCRP for Cardiovascular and Metabolic Diseases, South African Medical Research Council and University of Cape Town, Cape Town, South Africa; 4 Department of Medicine, McGill University, Montreal, Quebec, Canada; 5 Institute of Health and Society, Newcastle University, Newcastle, United Kingdom; Institute of Neuroepidemiology and Tropical Neurology, France

## Abstract

**Background and Purpose:**

Determinants of post-acute stroke outcomes in Africa have been less investigated. We assessed the association of metabolic syndrome (MetS) and insulin resistance with post-stroke mortality in patients with first-ever-in-lifetime stroke in the capital city of Cameroon (sub-Saharan Africa).

**Methods:**

Patients with an acute first-stroke event (n = 57) were recruited between May and October 2006, and followed for 5 years for mortality outcome. MetS definition was based on the Joint Interim Statement 2009, insulin sensitivity/resistance assessed via glucose-to-insulin ratio, quantitative insulin sensitivity check index and homeostatic model assessment.

**Results:**

Overall, 24 (42%) patients deceased during follow-up. The prevalence of MetS was higher in patients who died after 28 days, 1 year and 5 years from any cause or cardiovascular-related causes (all *p≤*0.040). MetS was associated with an increased overall mortality both after 1 year (39% vs. 9%) and 5 years of follow-up (55% vs. 26%, *p* = 0.022). Similarly, fatal events due to cardiovascular-related conditions were more frequent in the presence of MetS both 1 year (37% vs. 9%) and 5 years after the first-ever-in-lifetime stroke (43% vs. 13%, *p* = 0.017). Unlike biochemical measures of insulin sensitivity and resistance (non-significant), in age- and sex-adjusted Cox models, MetS was associated with hazard ratio (95% CI) of 2.63 (1.03–6.73) and 3.54 (1.00–12.56) respectively for all-cause and cardiovascular mortality 5 years after stroke onset.

**Conclusion:**

The Joint Interim Statement 2009 definition of MetS may aid the identification of a subgroup of black African stroke patients who may benefit from intensification of risk factor management.

## Introduction

Metabolic syndrome (MetS) is a constellation of conditions which singly are associated with increased risk of cardiovascular diseases (CVD) [1], [2]. Furthermore, the presence of MetS in an individual confers a risk of cardiovascular disease higher than that from each of the components of the syndrome [3], [4]. Therefore, MetS has been intensively investigated over the recent years for a possible contribution to cardiovascular disease risk stratification and/or reduction.

The prevalence of MetS varies substantially across populations and settings, both as a result of background differences in the distribution of its components across populations, but also of the diversity of criteria for defining the condition. There have been recent efforts to harmonize the clinical definition of MetS by accounting for ethnic differences in the cutoff values of key components such as central obesity and atherogenic dyslipidemia [5], [6]. How the harmonized definition reflects the risk of major outcomes has not been widely assessed. For instance, in the absence of cutoff values specific to populations of African ethnicity, those derived from Caucasians have been recommended in Africans, yet no evidence is available on the correlation of MetS based on those criteria and major incident health outcomes among black Africans [7]–[10]. Stroke is the most common cardiovascular outcome in sub-Saharan Africa and may serve this purpose.

We therefore sought to investigate the association of MetS with post-event survival in a cohort of patients following a first-ever-in-lifetime stroke from an urban area of Cameroon.

## Methods

### Study Setting and Participants

**Figure 1:**
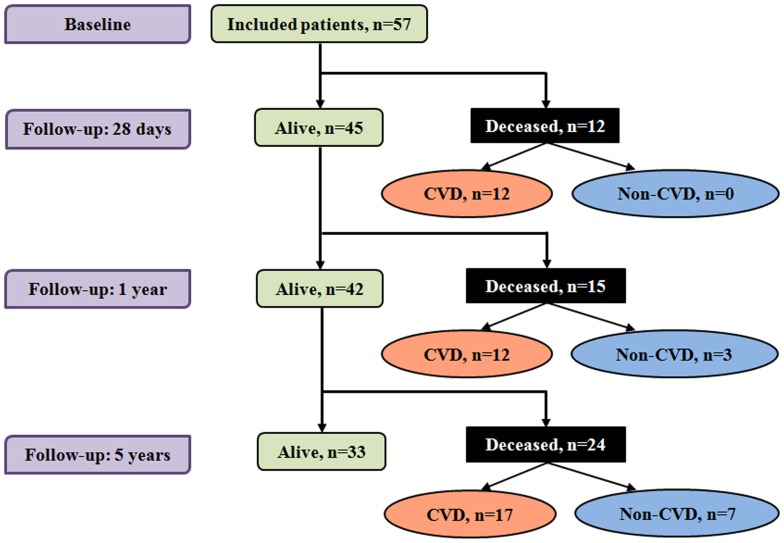
Flow chart of enrolled patients. Values are cumulative number of participants alive (green boxes) or who died (black boxes) at 28 days, 1 and 5 years of follow-up. The number of deaths at each time-point is further distinguished as cardiovascular disease (CVD) related (light-salmon ovals) or not (light-blue ovals).

This study was conducted at Yaoundé Central Hospital, a major tertiary reference hospital in the capital city of Cameroon. The setting and the study population have been previously described [11]. Briefly, patients admitted for a first-ever-in-lifetime stroke were consecutively enrolled between May and October 2006. Diagnosis of stroke was based on WHO criteria [12] and stroke was distinguished from transient ischemic attack by the duration of functional impairment or symptoms of more than 24 hours. Demographic, anthropometric and clinical data were collected at baseline using a standard questionnaire and blood samples were collected after an overnight fast for biological determinations. Waist circumference was measured in supine position since most patients were bedridden for major motor deficiency and could not assume the usual standing position required for such a measurement. Body mass index was calculated using the most recent measurement of height and weight in the last 12 months, in the absence of specific scale for measuring adult’s weight in supine position. Follow-up contacts were established with patients, their relatives and/or their physicians 28 days, 1 year and 5 years from baseline for mortality outcome data collection (Figure 1). Mortality and causes of death were ascertained via hospital records or verbal autopsy and based on the 10^th^ revision of the International Classification of Diseases (codes I 00–99) [13]. The study was approved by the Institutional Review Board of The Yaoundé Central Hospital (Cameroon). Patients, always in the presence of at least one relative, or two next of kin (for unconscious patients) provided the verbal informed consent to participate in the study. The purpose of the study was explained to participants/family in one of the official languages, or one of the national languages for illiterate patients, with the assistance of translators as appropriate. Informed verbal consent was deemed appropriate given the high illiteracy rate among patients with stroke and the nature of the disease with some patients presenting in unconscious or impotent states, and therefore unable to provide a written consent. The consent procedure was approved by the Institutional Review Board, and documented for each patient by a tick of boxes on the case report form, which was always presented to patients/families.

### Definition of Metabolic Syndrome

MetS was defined based on the Joint Interim Statement (JIS) criteria [5]. Thus, positive diagnosis of the syndrome was established when at least three of the following were present: 1) fasting plasma glucose ≥100 mg/dL (or history of doctor-diagnosed diabetes), 2) systolic (and/or diastolic) blood pressure ≥130 (85) mmHg or treated hypertension, 3) serum triglycerides ≥150 mg/dL, 4) serum high-density lipoproteins (HDL) cholesterol <40 mg/dL in men and <50 mg/dL in women and 5) waist circumference of >80 cm in women and >94 cm in men. Blood pressure was measured on the left arm using a mercury sphygmomanometer with appropriate cuffs and the average of two measurements taken 5 minutes apart was used in the study.

### Analytical Methods

Glucose was analyzed by the hexokinase method (Roche Diagnostics, Mannheim, Germany). Insulin levels were determined by radioimmunoassay (Linco Research; St. Charles, MO), while triglycerides (TG), total cholesterol (TC) and HDL cholesterol determinations used enzymatic colorimetric methods. A more detailed description of the analytical methods is available elsewhere [11]. Since none of the patients had a triglycerides level of 400 mg/dL or more, LDL cholesterol levels were calculated using Friedewald formula for all study participants [14]. Blood samples were collected between 72 hours and at most 1 week post-admission after an overnight fast of 12 hours.

Insulin sensitivity was assessed by both fasting glucose-to-insulin (Glu/Ins) ratio and quantitative insulin sensitivity check index (QUICKI) while insulin resistance was estimated by the Homeostatic model assessment of insulin resistance (HOMA-IR) three to seven days after admission [11].

### Statistical Analysis

Groups’ comparison used Chi-square or Fisher’s exact tests for categorical variables and student’s *t*-test or Mann-Whitney U test for continuous variables. Results are expressed as counts (proportions), mean and standard deviation or median, inter-quartile range, minimum and maximum values. Early (28-day), one- and five-year mortality rates were derived with the use of the Kaplan-Meier estimator, with groups comparisons via Log-rank test. Cox proportional hazard regression models were then used to adjust for the effect of age and sex, with the non-violation of the proportional assumption confirmed via Schoenfeld residuals’ plots. Follow-up duration was estimated from the day of hospital admission to death, lost to follow-up or maximum duration of 5 years, whichever came first. Because not all included patients (n = 31/57, 54%) could afford a CT-scan, we performed a sensitivity analysis and extrapolated our analysis to patients clinically diagnosed with ischemic stroke (n = 45). Clinical classification of stroke subtypes resulted in a 92% sensitivity and 60% specificity [11]. One participant was excluded from the survival analysis for missing data on MetS status. A *p* value <0.05 was used to characterize statistically significant results. SPSS v20.0 for Windows (IBM statistics, Chicago, IL, USA) and GraphPad Prism v5.00 for Windows (San Diego, CA, USA) were used for statistical analysis.

## Results

### Baseline Profile of the Study Group

The general characteristics of the study population are shown in Table 1. Of the 57 participants included, 32 (56%) were men. With the exception of high waist circumference which was more frequent in women than in men (72% vs. 38%), there was no significant difference in the distribution of baseline characteristics between men and women (all p≥0.105, Table 1). Stroke was clinically classified as ischemic in 45 (79%) patients and as hemorrhagic in 12 (21%) patients (Table 1). Imaging studies (CT scan) were available for 31 patients (54%) among whom 26 (84%) had ischemic stroke and 5 (16%) had hemorrhagic stroke [11]. Low HDL cholesterol (69%) was the most frequent component of MetS followed by high blood pressure (65%) and high waist circumference 53%. The prevalence of MetS was 59% (n = 33) overall, 50% (n = 16) in men and 71% (n = 17) in women (p = 0.196, Table 1).

**Table 1:** General characteristics of the study population at inclusion.

Characteristics	All patients (n = 57)	Men (n = 32)	Women (n = 25)	*p*
Age, years	61.9±12.9	63.1±10.4	60.4±15.6	0.439
Body mass index, kg/m^2^	23.7±11.5	25.4±10.6	19.6±13.0	0.160
Waist girth, cm	90.5±14.0	92.1±15.5	88.3±11.5	0.318
Systolic blood pressure, mmHg	170±36	171±37	168±35	0.709
Diastolic blood pressure, mmHg	101±27	106±33	94±15	0.105
Total cholesterol, mg/dL	173±36	169±40	178±39	0.389
Triglycerides, mg/dL	129±56	135±57	122±55	0.411
LDL cholesterol, mg/dL	109±43	106±40	114±47	0.483
HDL cholesterol, mg/dL	37±21	36±14	39±28	0.528
Fasting blood glucose, g/L	1.33±0.79	1.20±0.51	1.47±1.03	0.205
Plasma insulin, mIU/L	5.86±5.37	5.2±3.3	6.7±7.4	0.316
Clinical type of stroke				
Hemorrhagic, n (%)	12 (21)	7 (22)	6 (24)	>0.99
Ischemic, n (%)	45 (79)	25 (78)	20 (80)	>0.99
Components of MetS				
High blood pressure, n (%)	37 (65)	22 (69)	15 (60)	0.684
Diabetes, n (%)	26 (47)	15 (50)	11 (44)	0.657
High waist girth, n (%)	30 (53)	12 (38)	18 (72)	0.010
Low HDL cholesterol, n (%)	38 (69)	19 (59)	19 (83)	0.123
High triglyceride, n (%)	22 (40)	14 (44)	8 (35)	0.696
Metabolic syndrome, n (%)	33 (59)	16 (50)	17 (71)	0.196
Number of components, n (%)				
1	8 (14)	5 (16)	3 (13)	>0.99
2	15 (27)	11 (34)	4 (17)	0.223
3	20 (36)	10 (31)	10 (42)	0.601
4 or 5	13 (24)	6 (19)	7 (30)	0.494

### Fatal Outcomes

Of the 57 patients enrolled 33 (58%) were still alive after 5 years of follow-up. Table 2 summarizes the general characteristics of survivors and patients who died at all follow-up time-points. Men and women were equally distributed across all the study groups. Stroke survivors overall were mostly comparable to those who died with consideration to several baseline characteristics and at any given time-point during follow-up. Although they tended to be younger, leaner, to have lower fasting blood glucose, insulin, triglycerides, HDL cholesterol and LDL cholesterol levels, and to display lower systolic and diastolic blood pressures than those who died, differences did not reach the conventional significance threshold (Table 2). Similar findings were observed in the subset of patients clinically diagnosed with ischemic stroke (Table S1).

**Table 2:** Baseline characteristics of the study population according to mortality at different follow-up time-points.

Characteristics	Short term overall mortality				1-year overall mortality			5-year overall mortality			5-year cardiovascular-related mortality		
	Yes(n = 12)		No(n = 45)	*p*	Yes(n = 15)	No(n = 42)	*p*	Yes(n = 24)	No(n = 33)	*p*	Yes(n = 17)	No(n = 40)	*p*
Age, years	68±12		60±12	0.086	66±13	60±13	0.135	65±12	59±13	0.092	64.3±13.1	60.9±12.8	0.368
Sex ratio, men/women	5/7		27/18	0.255	7/8	25/17	0.389	13/11	19/14	0.798	8/9	24/16	0.542
Body mass index, kg/m^2^	25±13		23±11	0.718	27±12	23±11	0.353	26±13	23±11	0.477	26.9±11.0	22.7±11.7	0.348
Waist girth, cm	92±10		90±15	0.655	94±13	89±14	0.265	93±13	89±15	0.267	94.9±11.9	88.5±14.5	0.114
Systolic blood pressure, mmHg	159±37		173±36	0.246	159±36	174±36	0.185	163±35	174±36	0.242	164±35	172±37	0.488
Diastolic blood pressure, mmHg	90±15		104±29	0.130	91±16	104±30	0.127	94±15	106±33	0.094	94±15	104±31	0.212
Total cholesterol, mg/dL	185±36		169±40	0.229	173±41	172±39	0.941	170±39	175±41	0.664	171±37	173±41	0.893
Triglycerides, mg/dL	132±50		129±58	0.842	136±54	127±57	0.599	126±56	132±56	0.675	122±48	132±60	0.527
LDL cholesterol, mg/dL	125±41		105±43	0.171	113±45	108±43	0.714	112±43	107±44	0.714	112±41	108±44	0.765
HDL cholesterol, mg/dL	34±17		38±21	0.516	33±17	39±22	0.362	33±15	41±24	0.168	35±17	38±22	0.591
Fasting blood glucose, g/L	1.62±0.99		1.25±0.72	0.159	1.49±0.91	1.26±0.75	0.355	1.36±0.79	1.31±0.81	0.815	1.46±0.88	1.27±0.76	0.422
Plasma insulin, mIU/L	5.0±2.6		6.1±5.9	0.533	4.8±2.7	6.3±6.0	0.374	5.6±5.5	6.0±5.3	0.775	6.6±6.1	5.5±5.0	0.506
Components of MetS													
High blood pressure, n (%)		9 (75)	28 (62)	0.510	11 (73)	26 (62)	0.537	18 (75)	19 (56)	0.174	13 (77)	24 (60)	0.374
Diabetes, n (%)		8 (67)	18 (42)	0.128	9 (60)	17 (43)	0.247	13 (54)	13 (42)	0.368	10 (59)	16 (42)	0.392
High waist girth, n (%)		8 (67)	22 (49)	0.340	10 (67)	20 (48)	0.205	15 (62)	15 (45)	0.203	12 (71)	18 (45)	0.139
Low HDL cholesterol, n (%)		9 (75)	29 (67)	0.735	11 (73)	27 (67)	0.754	18 (75)	20 (64)	0.404	12 (32)	26 (68)	>0.99
High triglyceride, n (%)		5 (42)	17 (39)	0.894	6 (40)	16 (40)	1.00	8 (33)	14 (45)	0.375	5 (29)	17 (45)	0.439
Metabolic syndrome, n (%)		11 (92)	22 (50)	0.010	13 (87)	20 (49)	0.014	18 (75)	15 (47)	0.034	14 (82)	19 (49)	0.040
Number of components, n (%)													
1		1 (8)	7 (16)	0.672	1 (7)	7 (17)	0.428	3 (12)	5 (16)	1.00	2 (12)	6 (15)	>0.99
2		0 (0)	15 (34)	0.024	1 (7)	14 (34)	0.047	3 (12)	12 (37)	0.065	1 (6)	14 (36)	0.023
3		6 (50)	14 (31)	0.313	8 (53)	12 (29)	0.096	10 (42)	10 (31)	0.421	8 (47)	12 (31)	0.386
4 or 5		5 (42)	8 (19)	0.096	5 (33)	8 (20)	0.300	8 (33)	5 (16)	0.136	6 (35)	7 (18)	0.190

### Metabolic Syndrome and Insulin Resistance

The distribution of individual components of MetS was similar between survivors and deceased participants at any time-point. However, the prevalence of JIS-defined MetS was always higher among deceased patients. Prevalence figures (deceased vs. survivors) were 11/12 vs. 22/45 at 28 days, 13/15 vs. 20/42 at 1 year, 18/24 vs. 15/33 at 5 years for all-cause mortality, and 14/17 vs. 19/40 at 5 years for cardiovascular-related death (all *p≤*0.04, Table 2). No difference in the prevalence of individual components of MetS was observed although deceased patients from cardiovascular-related causes at 5-year among those with clinically diagnosed ischemic stroke had a higher waist circumference (Table S1).

**Figure 2:**
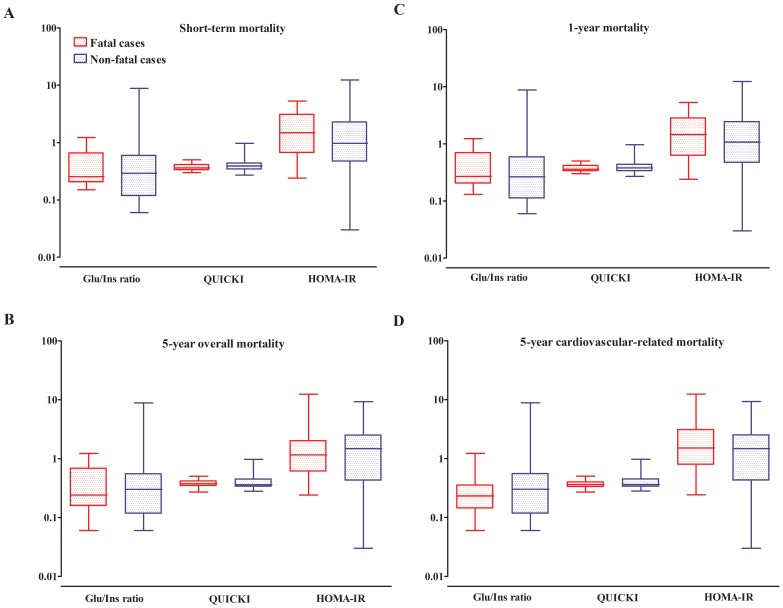
Insulin sensitivity and resistance according to outcome and cause of death during follow-up. Comparison of insulin resistance and sensitivity indices according to the vital status at 28 days (A), 1 year (B), 5 years from all-cause of death (C) and 5 years cardiovascular-related mortality (D). Data are expressed in a log scale. Boxes represent median (crossing horizontal bar) and interquartile range (lower and upper limits). The whiskers depict maximum and minimum values for each of the insulin resistance/sensitivity indices for deceased patients (red color) and those still alive (blue color) at the indicated follow-up time. For each insulin resistance/sensitivity index, and across figure panels, the boxes and whiskers for the deceased are always displayed on the right.

As previously mentioned, HOMA-IR was used to evaluate insulin resistance while QUICKI and the Glu/Ins ratio were used as surrogates of sensitivity to insulin. Figure 2 shows that there was no significant difference between stroke survivors and those who died with regard to the levels of those predictors of insulin response. Although deceased patients tended to be more insulin-resistant (and less insulin-sensitive) than their counterparts, overall, no significant difference was observed in insulin sensitivity (glucose-to-insulin ratio and QUICKI) and resistance indices (HOMA-IR) between the two groups.

### Kaplan Meier and Cox Regression Analysis

**Figure 3:**
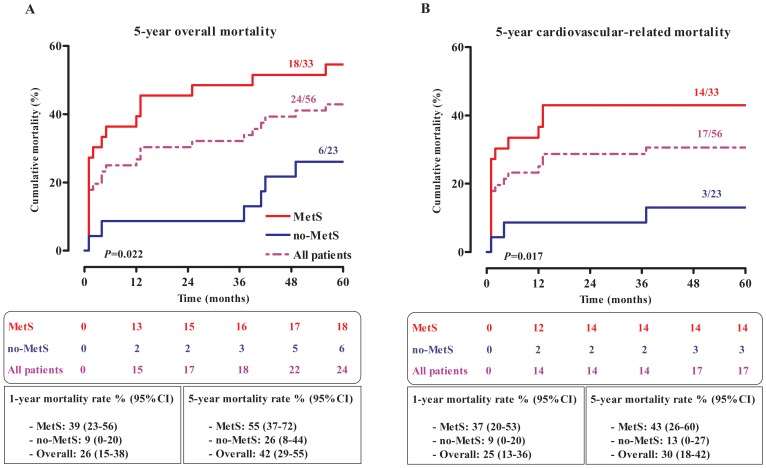
Kaplan-Meier curves in all included patients. Overall (A) and cardiovascular-related (B) mortalities according to the presence of metabolic syndrome. Comparisons were performed using Log-rank test. The total fraction of deceased patients during follow-up is indicated above each arm in the 2 panels. Cumulative number of deceased patients during follow-up and mortality rates (95%CI) after 1 and 5 years from overall and cardiovascular-related causes for all patients and in the presence or absence of MetS are indicated below the respective graphs.

Mortality rate from all causes and from cardiovascular-related causes in patients with first-ever-in-lifetime stroke was respectively 43% (95% CI, 30–56%) and 31% (95% CI, 18–43%) after 5 years. This and the stratification according to presence of MetS are illustrated in Figure 3. Overall mortality occurred more often in patients with MetS (Log-rank test, *p* = 0.022; Figure 3A). The latter group had a higher mortality rate both after 1 and 5 years follow-up (39% vs. 9% and 55% vs. 26% respectively). Survival analysis in patients who died from cardiovascular-related conditions suggests that 14/17 (82%) of deaths occurred within 12 months and the majority of deceased patients 14/17 (82%) had MetS. Mortality rates associated to MetS at 1 and 5 years were respectively 37% (20–53%) and 43% (26–60%). The 5-year mortality rate was significantly higher in the presence of MetS than in its absence (*p* = 0.017, Figure 3B). When patients with ischemic stroke were considered, 12/17 (71%) of deceased patients within 5 years had MetS which was associated with a 50% (95%CI, 30–70%; data not shown) all-cause mortality rate as opposed to 25% (95%CI, 6–44%; data not shown) among their counterparts without MetS (*p* = 0.047). In this subset of the study population, cardiovascular-related mortality occurred significantly earlier in the presence of MetS, 42 (95%CI, 22–62%; data not shown) vs. 10% (95%CI, 0–23%; data not shown), *p* = 0.017 (Figure S1).

In age- and sex-adjusted Cox regression analysis, the presence of MetS was associated with a hazard ratio (95% CI) of 2.63 (1.03–6.73), *p* = 0.043 and 3.54 (1.00–12.56), *p* = 0.050 respectively for all-cause and cardiovascular-related mortality after five years. Markers of insulin sensitivity or insulin resistance were not associated with 5-year mortality (all *p*>0.05, Table 3). Similarly, none of the components of MetS taken separately was significantly associated with mortality risk (data not shown). In patients with ischemic stroke, none of the candidate markers reached statistical significance for the prediction of overall mortality (all *p*>0.05) while MetS tended to be associated with 5-year mortality from cardiovascular-related conditions (*p* = 0.048, Table S2).

**Table 3:** Cox regression analysis of 5-year mortality in all study participants.

	5-year overall mortality			5-year cardiovascular-related mortality		
Covariates	HR*	95%CI	*p*	HR	95%CI	*p*
Glu/Ins ratio†	0.81	0.42–1.55	0.527	0.68	0.24–1.97	0.480
HOMA-IR‡	1.04	0.88–1.22	0.673	1.08	0.92–1.26	0.371
QUICKI§	0.06	0–16.88	0.331	0.10	0–10.36	0.171
Metabolic syndrome	2.63	1.03–6.73	0.043	3.54	1.00–12.56	0.050

All Cox models are adjusted for age and gender;

* hazard ratio;

† glucose-to-insulin ratio;

‡ homeostatic model assessment of insulin resistance;

§ quantitative insulin sensitivity check index.

## Discussion

We have investigated the relationship between post-stroke mortality and MetS using the recent definition criteria by the Joint Interim Statement for harmonization of MetS [5]. In our cohort, the prevalence of MetS but not its components was higher in stroke patients who died after 28 days, 1 and 5 years even after adjustment for age and gender. This was observed for both all-cause and cardiovascular-related mortality but with a less robust association with the latter.

Previous studies of MetS in sub-Saharan Africa used a variety of definitions including the ones proposed by the International Diabetes Federation, the World Health Organization (WHO) and the National Cholesterol Education Panel (NCEP) Adult Treatment Panel III [15]. The prevalence of MetS varies according to the criteria used for its definition in various ethnic groups and the JIS definition may result in a better estimate of the syndrome in sub-Saharan populations [5], [16], [17]. We found that hypertension and low HDL cholesterol were the most prevalent components of MetS in both survivors and deceased patients without significant differences between the two groups for all components. Moreover, MetS was more prevalent among fatal stroke cases. This suggests that risk of stroke onset at baseline from the individual risk factors in all patients is the same but they tend to occur concomitantly and therefore increase mortality rate in deceased patients. Similar findings have been described in African-Caribbean population with stroke and coronary heart disease [18].

Unlike insulin resistance or sensitivity indices, in the presence of MetS, patients with ischemic stroke died significantly faster independently of the cause of death. Overall, MetS was associated with a higher mortality rate and was an independent predictor of 5-year post-stroke death independently of the cause. A similar trend was observed for deaths from cardiovascular-related conditions. This finding is consistent with previously reported data using other definitions of MetS. In a logistic regression analysis and using WHO criteria for diagnosis of MetS, Isomaa et al. reported a 1.81 relative risk of death from cardiovascular-related events over a follow-up duration of 6.9 years [19]. Other significant predictors in the study include hypertension and microalbuminuria. The latter has not been assessed in our study and the former is estimated by the WHO criteria using a higher cutoff for blood pressure. Similarly, other studies defining MetS according to the NCEP or WHO have shown an increased mortality associated with the condition [4], [20], [21]. More importantly, it is apparent from our results that cardiovascular mortality occurs more often few months after stroke onset in patients with MetS. This suggests that targeting MetS might be effective for prevention of early mortality associated to cerebrovascular events. However, it is worthwhile to notice that our observations are based on a model restricted to metabolic parameters. The interplay between MetS and other variables such as stroke severity or the stroke subtype might influence the resulting outcome.

Unlike the pathophysiology of cardiovascular events in individuals with MetS, the relationship between MetS and mortality following cardiovascular events in general and stroke in particular still needs to be clarified [22]. The differences in definition and heterogeneity in populations of interest in studies focusing on MetS make the findings difficult to pool together. For instance, in some reports, participants were enrolled based on diabetes status, gender or age and in others not [2], [3], [23]–[25]. Nevertheless, in recent meta-analysis, both Galassi et al. and Gami et al. have shown that independently of these differences, MetS is associated with an increased risk of death following a cardiovascular event [20], [26]. Whether or not this excess mortality risk is due to the constellation of factors included in the definition of MetS is still unclear. Consistent with what has been described in other settings, our findings suggest that MetS is more prevalent and might predict poor outcome in patients with first-ever-in-lifetime stroke. However, no further inference about the relationship between the number of risk factors and mortality can be made due to the relatively small sample size.

The small sample size is the major limitation of this study. This may have affected our capacity for detecting some significant differences and precluded stratification according to stroke subtypes. Moreover, in the absence of systematic CT-Scan or magnetic resonance imaging studies, the definitive diagnosis of the subtypes of stroke was not available for all participants. Some patients with acute stroke would tend to die prior to hospital admission in this setting; therefore our use of hospital-based cohort would underestimate the true magnitude of early post-stroke mortality. We also lack data on medication prescription and adherence during follow-up, which could aid the understanding of some of the associations observed in this study. Lastly, waist girth was measured in supine position, and BMI calculated based on imputed weight using the most recent measurement in the last 12 months. Both approaches are imprecise, but are among the best alternatives for approximating those parameters in bedridden patients in our resources-limited setting. Our study also has major advantages including extensive baseline investigation, and a successful follow-up of a relatively large number of participants, which is rather uncommon in the African setting [8]. By demonstrating a correlation between JIS-defined MetS and long-term outcome, we have provided evidence in support of the applicability of this definition, although larger studies will be needed to assess the validity of individual components of the syndrome based on JIS definition in this context.

In conclusion, JIS-defined MetS is associated with an increased cardiovascular-related and all-cause mortality in black African patients with first stroke event. MetS therefore appears as a useful tool for identifying a subgroup of patients with stroke whose long-term outcomes can potentially be improved by more intensive risk factor modifying therapies. However, our observations need to be confirmed in a bigger cohort and the process by which the combination of risk factors in MetS influences case fatality warrants further investigation. In such a cohort, further investigation of the relationship between MetS as defined by the JIS 2009 [5] criteria and stroke severity using currently available scores could also be addressed.

## Supporting Information

Figure S1 **Kaplan-Meier curves in the subgroup of patients clinically diagnosed with ischemic stroke.** Overall (A) and cardiovascular-related (B) mortalities according to the presence of metabolic syndrome. Comparisons were performed using Log-rank test. The total fraction of deceased patients during follow-up is indicated above each arm in the 2 panels. Cumulative numbers of deceased patients during follow-up in the overall cohort of patients with ischemic stroke and in the presence or absence of MetS are mentioned below the panels.
(TIF)

Table S1 **Baseline characteristics of study participants with ischemic stroke according to mortality at different follow-up time-points.**
(DOCX)

Table S2 **Cox regression analysis for prediction of 5-year mortality in study participants with ischemic stroke.**
(DOCX)
