# Correction: Metabolic Syndrome and Fatal Outcomes in the Post-Stroke Event: A 5-Year Cohort Study in Cameroon

**DOI:** 10.1371/annotation/8410c942-8d5a-44c7-83e0-dc6ffe6b7c85

**Published:** 2013-12-17

**Authors:** Eric Vounsia Balti, André Pascal Kengne, Jean Valentin Fogha Fokouo, Brice Enid Nouthé, Eugene Sobngwi

In Figure 3A, "no-MetS" month 36 should show n=2. In Figure 3B, "All patients" month 24 should show n=16, and "All patients" month 36 should show n=16. Please see the corrected Figure 3 here: http://plosone.org/corrections/pone.0060117.g003.cn.tif

