# Supplementary material for: Metabolic Syndrome and Fatal Outcomes in the Post-Stroke Event: A 5-Year Cohort Study in Cameroon
Source: PLoS One. 2013 Apr 2;8(4):e60117. doi: 10.1371/journal.pone.0060117 (PMC3615065; doi:10.1371/journal.pone.0060117)
Supplement: Table S1 — Baseline characteristics of study participants with ischemic stroke according to mortality at different follow-up time-points. (DOCX) [file pone.0060117.s002.docx]

|  | | **Short term overall mortality** | | | **1-year overall mortality** | | | **5-year overall mortality** | | | **5-year cardiovascular-related mortality** | | |
| --- | --- | --- | --- | --- | --- | --- | --- | --- | --- | --- | --- | --- | --- |
| **Characteristics** | | **Yes (n=8)** | **No (n=37)** | ***p*** | **Yes (n=1)** | **No (n=34)** | ***p*** | **Yes (n=1)** | **No (n=28)** | ***p*** | **Yes (n=1)** | **No (n=33)** | ***p*** |
| Age, years | | 67.8±13.7 | 60.2±13.1 | 0.151 | 65.8±14.8 | 60.2±13.0 | 0.229 | 65.4±12.9 | 59.3±13.2 | 0.141 | 63.7±14.9 | 60.8±12.8 | 0.533 |
| Gender, men/women | | 2/6 | 23/14 | 0.113 | 4/7 | 21/13 | 0.176 | 9/8 | 16/12 | 1.00 | 5/7 | 20/13 | 0.429 |
| Body mass index, kg/m^2^ | | 30.6±6.8 | 23.2±12.3 | 0.320 | 31.4±6.2 | 22.5±12.4 | 0.131 | 27.6±12.5 | 22.4±11.7 | 0.286 | 30.3±6.1 | 22.3±12.6 | 0.148 |
| Waist girth, cm | | 93.8±9.9 | 89.9±13.9 | 0.470 | 95.8±13.0 | 88.9±13.1 | 0.137 | 94.1±13.4 | 88.5±13.0 | 0.178 | 98.0±11.4 | 87.9±12.6 | 0.022 |
| Systolic blood pressure, mmHg | | 150±34 | 168±33 | 0.172 | 153±35 | 169±33 | 0.167 | 156±35 | 171±32 | 0.153 | 161±36 | 167±33 | 0.630 |
| Diastolic blood pressure, mmHg | | 87±17 | 100±21 | 0.102 | 90±18 | 100±20 | 0.137 | 93±17 | 101±22 | 0.221 | 94±17 | 99±21 | 0.449 |
| Total cholesterol, mg/dL | | 196±36 | 167±39 | 0.063 | 177±45 | 171±38 | 0.658 | 172±43 | 173±38 | 0.924 | 178±40 | 170±40 | 0.616 |
| Triglycerides, mg/dL | | 162±30 | 128±51 | 0.078 | 159±49 | 126±43 | 0.054 | 143±49 | 129±49 | 0.341 | 145±37 | 130±53 | 0.393 |
| LDL cholesterol, mg/dL | | 128±44 | 103±43 | 0.150 | 111±48 | 106±43 | 0.750 | 109±46 | 106±43 | 0.831 | 111±44 | 106±45 | 0.701 |
| HDL cholesterol, mg/dL | | 36±18 | 39±23 | 0.740 | 35±18 | 40±23 | 0.499 | 34±16 | 41±25 | 0.308 | 37±18 | 39±24 | 0.807 |
| Fasting blood glucose, g/L | | 1.81±1.13 | 1.31±0.78 | 0.139 | 159±102 | 134±81 | 0.407 | 141±90 | 139±86 | 0.934 | 1.58±0.99 | 1.33±0.82 | 0.414 |
| Plasma insulin, mIU/L | | 4.7±3.1 | 6.7±6.2 | 0.397 | 4.5±2.9 | 6.9±6.4 | 0.241 | 60.±6.4 | 6.6±5.5 | 0.754 | 6.9±7.3 | 6.1±5.3 | 0.707 |
| Components of MetS | | |  |  |  |  |  |  |  |  |  |  |  |
|  | High blood pressure, n (%) | 5 (63) | 21 (57) | 1.00 | 7 (64) | 19 (56) | 0.736 | 11 (65) | 15 (54) | 0.673 | 8 (67) | 18 (55) | 0.699 |
|  | Diabetes, n (%) | 6 (75) | 15 (43) | 0.132 | 7 (64) | 14 (44) | 0.430 | 9 (53) | 12 (46) | 0.902 | 8 (67) | 13 (42) | 0.265 |
|  | High waist girth, n (%) | 6 (75) | 16 (43) | 0.135 | 8 (73) | 14 (41) | 0.141 | 10 (59) | 12 (43) | 0.465 | 9 (75) | 13 (39) | 0.076 |
|  | Low HDL cholesterol, n (%) | 6 (75) | 24 (67) | 1.00 | 8 (73) | 22 (73) | 1.00 | 12 (71) | 18 (67) | 1.00 | 8 (67) | 22 (69) | >0.99 |
|  | High triglyceride, n (%) | 5 (63) | 14 (39) | 0.262 | 6 (55) | 13 (39) | 0.489 | 7 (41) | 12 (44) | 1.00 | 5 (42) | 14 (44) | >0.99 |
| Metabolic syndrome, n (%) | | 8 (100) | 16 (44) | 0.005 | 10 (91) | 14 (42) | 0.005 | 12 (71) | 12 (44) | 0.166 | 10 (83) | 14 (44) | 0.045 |
| Number of components, n (%) | | |  |  |  |  |  |  |  |  |  |  |  |
|  | 1 | 0 (0) | 6 (17) | 0.573 | 0 (0) | 6 (18) | 0.311 | 2 (12) | 4 (15) | 1.00 | 1 (8) | 5 (16) | >0.99 |
|  | 2 | 0 (0) | 14 (39) | 0.041 | 1 (9) | 13 (39) | 0.076 | 3 (18) | 11 (41) | 0.204 | 1 (8) | 13 (41) | 0.068 |
|  | 3 | 4 (50) | 10 (28) | 0.242 | 6 (55) | 8 (24) | 0.132 | 7 (41) | 7 (26) | 0.468 | 5 (42) | 9 (28) | 0.475 |
|  | 4 or 5 | 4 (50) | 6 (17) | 0.064 | 4 (36) | 6 (18) | 0.237 | 5 (29) | 5 (18) | 0.473 | 5 (42) | 5 (16) | 0.105 |

**Table S1:** Baseline characteristics of study participants with ischemic stroke according to mortality at different follow-up time-points.
