# Supplementary material for: Metabolic Syndrome and Fatal Outcomes in the Post-Stroke Event: A 5-Year Cohort Study in Cameroon
Source: PLoS One. 2013 Apr 2;8(4):e60117. doi: 10.1371/journal.pone.0060117 (PMC3615065; doi:10.1371/journal.pone.0060117)
Supplement: Table S2 — Cox regression analysis for prediction of 5-year mortality in study participants with ischemic stroke. (DOCX) [file pone.0060117.s003.docx]

**Table S2:** Cox regression analysis for prediction of 5-year mortality in study participants with ischemic stroke.

|  | **5-year overall mortality** | | | **5-year cardiovascular-related mortality** | | |
| --- | --- | --- | --- | --- | --- | --- |
| **Covariates** | **HR^*^** | **95%CI** | ***p*** | **HR** | **95%CI** | ***p*** |
| Glu/Ins ratio^†^ | 0.85 | 0.46-1.58 | 0.616 | 0.81 | 0.36-1.84 | 0.619 |
| HOMA-IR^‡^ | 1.03 | 0.86-1.25 | 0.726 | 1.07 | 0.89-1.28 | 0.480 |
| QUICKI^§^ | 0.26 | 0.1-72.86 | 0.639 | 0.04 | 0-107.94 | 0.422 |
| Metabolic syndrome | 2.53 | 0.88-7.28 | 0.085 | 4.71 | 1.01-21.90 | 0.048 |

All Cox models are adjusted for age and gender; ^*^hazard ratio; ^†^glucose-to-insulin ratio; ^‡^homeostatic model assessment of insulin resistance; ^§^quantitative insulin sensitivity check index.
